# Plasma Cholesterol-Lowering Activity of Soybean Germ Phytosterols

**DOI:** 10.3390/nu11112784

**Published:** 2019-11-15

**Authors:** Hanyue Zhu, Jingnan Chen, Zouyan He, Wangjun Hao, Jianhui Liu, Erika Kwek, Ka Ying Ma, Yanlan Bi

**Affiliations:** 1 School of Life Sciences, Chinese University of Hong Kong, Shatin, Hong Kong 999077, China; zhuhanyue29@gmail.com (H.Z.); hezuoyan1017@gmail.com (Z.H.); hwjcuhk@hotmail.com (W.H.); joyce91ha@gmail.com (J.L.); erika.kwek@hotmail.com (E.K.); rubyma06@cuhk.edu.hk (K.Y.M.); 2 College of Food Science and Technology, Henan University of Technology, Zhengzhou 450000, China; bylzry@126.com

**Keywords:** soybean germ phytosterols, plasma cholesterol, cholesterol absorption, hamster

## Abstract

Soybean germ phytosterols (SGP) largely exist in soybean germ oil. Our previous study demonstrated that soybean germ oil was effective in reducing plasma cholesterol. However, it remains unknown if its phytosterols are the active ingredients responsible for the plasma cholesterol-lowering activity. The present study aimed to test the effect of SGP on plasma cholesterol and to investigate its associated underlying mechanisms using hamsters as animal model. Male hamsters (*n* = 40) were randomly divided into five groups (*n* = 8/group) and fed one of the five diets: a non-cholesterol diet (NCD), a high cholesterol diet (HCD), a HCD diet containing 0.5% cholestyramine (PC), and two HCD diets containing 0.1% (LP) and 0.2% (HP) SGP, respectively, for six weeks. Results showed that SPG reduced plasma cholesterol level in a dose-dependent manner, whereas it dose-dependently increased the excretion of both fecal neutral and acidic sterols. SGP was also effective in displacing cholesterol from micelles. It was concluded that SGP possessed hypocholesterolemic activity, likely by inhibiting cholesterol absorption in the intestine and promoting fecal sterol excretion.

## 1. Introduction

Hypercholesterolemia refers to high levels of total cholesterol (TC) and low-density lipoprotein cholesterol (LDL-C) in blood and is one of the major risk factors for coronary heart disease (CHD). Statins, cholestyramine and ezetimibe are the three types of major medicines used clinically to reduce plasma TC for patients whose blood TC is beyond 240 mg/dL or 6.2 mM. However, these drugs may cause various side effects [1,2,3]. Herbal medicine may therefore be an alternative in the treatment of hypercholesterolemia, particularly for patients whose blood cholesterol is marginally high (between 200–240 mg/dL or 5.2–6.2 mM) [4,5,6]. As a herbal supplement, dietary phytosterols have been demonstrated to reduce blood cholesterol in both humans and animals [7,8,9]. However, their exact mechanism of action is still inconclusive [10]. Some studies claim that phytosterols can regulate the transporter-mediated processes of cholesterol uptake, whereas others demonstrate that phytosterols are capable of competing with cholesterol for incorporation into micelles before absorption in the intestine [11,12,13].

Cholesterol absorption in the intestine requires several transporters and enzymes. To begin with, free cholesterol is packed into micelles facilitated by bile acids, which then enter enterocytes with the assistance of the sterol transporter Niemann-Pick C1-Like 1 (NPC1L1) [14]. Acyl-CoA: cholesterol acyltransferase 2 (ACAT2) is responsible for intracellular cholesterol esterification in enterocytes. Subsequently, microsomal triacylglycerol transport protein (MTP) facilitates the assembly of triacylglycerols and cholesteryl esters into chylomicrons before entering the lymphatic system [15]. To avoid the accumulation of excessive cholesterol in enterocytes, the un-esterified cholesterol returns to the lumen of the intestine by ATP binding cassette transporters sub-family G member 5 and 8 (ABCG5/8) [16]. Regarding cholesterol synthesis and elimination, numerous enzymes and proteins are required, including 3-hydroxy-3-sterol regulatory element binding protein 2 (SREBP2), 3-hydroxy-3 methylglutaryl-CoA reductase (HMG-CoA-R), LDL receptor (LDL-R) and cholesterol-7α-hydroxylase (CYP7A1). In the liver, SREBP2 controls the gene expression of HMG-CoA-R and LDLR in cholesterol synthesis and removal [17]. CYP7A1 is a key enzyme for converting cholesterol to bile acids for elimination [18].

Our previous study demonstrated that soybean germ oil was effective in reducing plasma TC [19]. However, it remains unknown if its phytosterols are the active ingredients contributing to the blood cholesterol-lowering activity of soybean germ oil. Accounting for 0.4% total germ weight, soybean germ phytosterols (SGP) are very unique because they contains eight isomers including campesterol, stigmasterol, β-sitosterol, ∆7-stigmastenol, ∆7-avenasterol and citrostadienol [20]. To the best of our knowledge, the management of hypercholesterolemia using SGP has not yet been reported. The aim of the present study was to investigate the effectiveness and mechanisms by which SGP reduced plasma cholesterol level.

## 2. Materials and Methods

### 2.1. SGP Preparation

Soybean germs were obtained from Riotto Botanical Co. Ltd. (Xi’an, China). Soybean germ oil was extracted from soybean germs as previously described [19]. To extract SGP from soybean germ oil, soybean germ oil (50 g) was refluxed and saponified in NaOH 90% ethanol solution (1 M, 500 mL) for 1 h. SGP was extracted into 100 mL n-hexane three times. Finally, the hexane was removed in a rotary evaporator under vacuum. The final SGP was weighed and the yield was 6.0%.

### 2.2. Diets

Five diets were prepared as previously described with some modifications [19]. The non-cholesterol diet (NCD) was prepared by mixing corn starch, casein, sucrose, lard, minerals, vitamins, gelatin and DL-methionine as shown in Table 1. The high-cholesterol diet (HCD) was similarly produced by adding 0.1% cholesterol into the NCD diet. The positive control diet (PC) was prepared by adding 0.5% cholestyramine into the HCD diet. Cholestyramine is a well-known cholesterol-lowering drug, which can effectively promote bile acid excretion. Two SGP diets were formulated by supplementing 0.1% (LP) and 0.2% (HP) SGP into the HCD diet (Table 1).

### 2.3. Hamsters

Forty male Golden Syrian Hamsters (aged 3 months, 100–120 g) were randomly divided into 5 groups (*n* = 8 each), and fed one of the five diets described above. At week 0 and 6 of feeding, 1.0 mL of blood was collected from the retro-orbital sinus of each hamster under a light anesthesia after overnight fasting. After feeding for 6 weeks, hamsters were sacrificed under CO_2_ anesthesia. The organs including the liver, heart, kidney, testis, intestine, perirenal fat pad, and epididymal fat pad were dissected and weighed. The entire protocol was approved by the Experimental Animal Ethics Committee, The Chinese University of Hong Kong.

### 2.4. Analysis of Phytosterols

The phytosterol profile of SGP was characterized using a gas chromatography (GC) method [17,21]. In brief, phytosterols in SGP were converted to their tetramethylsilane (TMS) derivatives. The individual phytosterol TMS derivatives were analyzed on a fused silica capillary column (SACTM-5, 30 m × 0.25 mm, i.d.; Supelco, Inc., Bellefonte, PA, USA) in a Shimadzu GC-2010 GC equipped with a flame ionization detector (Tokyo, Japan). The individual phytosterols were identified by comparing the retention times of corresponding authentic standards.

### 2.5. Measurement of Plasma Lipoproteins

Plasma TC, high-density lipoprotein cholesterol (HDL-C) and triacylglycerols (TG) were measured using commercial enzymatic kits from Stanbio Laboratories (Boerne, TX, USA). Non-HDL-C was calculated by subtracting HDL-C from TC.

### 2.6. Analysis of Atherosclerotic Plaque

To assess the effect of SGP on atherosclerosis, the fatty plaque area on the thoracic aorta was measured using a previously described method [22]. In brief, the aorta was carefully dissected, then opened and stained with saturated oil red. The atherosclerotic plaque area in stained aorta was then scanned and quantified.

### 2.7. Determination of Liver Cholesterol

To assess the effect of SGP on liver cholesterol, a GC method was used [23]. First, total liver lipids were extracted using a solvent mixture of chloroform–methanol. Second, the total liver lipids were saponified and cholesterol was converted to its TMS derivative followed by quantification on a SAC^TM−5^ column in a Shimadzu GC-2010 GC equipped with a flame ionization detector. 5α-Cholestane was used as an internal standard.

### 2.8. Measurement of Fecal Sterols

To assess the effect of SGP on the cholesterol absorption and excretion, a GC method was used to quantify total fecal sterols [24,25]. Fecal samples were freeze-dried and powdered. 5α-Cholestane and hyodeoxycholic acid were added into weighed fecal samples as internal standards to quantify neutral sterols and fecal bile acids, respectively. After saponification and extraction, these fecal sterols were converted to their corresponding TMS-ether derivatives for further GC analysis. Each sample was measured in duplicate.

### 2.9. Real-Time PCR and Western Blot Analyses

To assess the interaction of SGP with gene expression related to cholesterol absorption in the intestine and cholesterol metabolism in the liver, the mRNA and protein mass of the following proteins were quantified: NPC1L1, ABCG5/8, ACAT2, MTP, SREBP2, LDLR, HMG-CoA-R, CYP7A1, and liver X receptor alpha (LXRα) as previously described [16]. In brief, total RNA was extracted and then converted to complementary DNA. Real-time PCR analysis was carried out on a Step One Plus Real-Time PCR System. The target proteins were separated on a 7% SDS-PAGE gel and transferred onto polyvinylidene difluoride membranes using a semi-dry transfer system. Membranes were blocked in 3% non-fat milk or 3% bovine serum albumin (BSA), followed by incubation for 12 h with their respective primary antibodies. Then, the membranes were incubated with the secondary antibodies for 1 h. Protein bands were analyzed using Touch Imaging System Software and β-actin was selected to normalize the protein mass.

### 2.10. Displacing Effect of SGP on Cholesterol in Micelles

The effect of SGP on the incorporation of cholesterol in micelles was assessed using an in vitro method [10]. In brief, the basal micellar solution was prepared by mixing oleic acid (0.39 mM), taurocholate acid (5 mM) and monoolein (0.11 mM) into 15 mM sodium phosphate buffer (pH = 7.4) containing 132 mM NaCl. Three micellar solutions were prepared: LC, adding 0.25 mM cholesterol into the micellar solution; HC, adding 0.50 mM cholesterol into the micellar solution; and SGP, adding 0.25 mM cholesterol and 0.25 mM SGP into the micellar solution. These three micellar solutions were sonicated for 0.5 h at 37 °C and then ultracentrifuged at 100,000 × *g* for 1 h at 37 °C. The bottom precipitate was discarded. The amount of cholesterol and SGP in the upper clear micellar phase was extracted and measured using the GC method described above.

### 2.11. Statistics

All data were analyzed by one-way analysis of variance (ANOVA) followed by Fisher’s Least Significant Difference (LSD) test. Significant differences across the five groups were defined as a *p* value < 0.05.

## 3. Results

### 3.1. Phytosterol Composition

**Figure 1:**
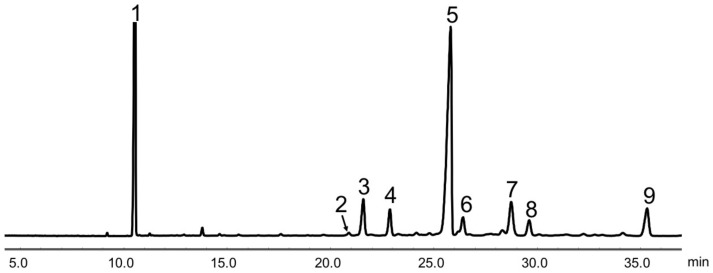
Gas chromatogram of soybean germ phytosterols (SGP). Peaks: 1, 5α-cholestane (internal standard); 2, ergosterol; 3, campesterol; 4, stigmasterol; 5, β-sitosterol; 6, stigmastanol; 7, ∆7-stigmastenol; 8, ∆7-avenasterol; 9, citrostadienol.

GC analysis showed that the purity of SGP used in the present study was about 80%. It was mainly made up of β-sitosterol (60.0%), ∆7-stigmastenol (9.8%), citrostadienol (7.4%), campesterol (7.2%), stigmasterol (4.8%), stigmastanol (4.7%) and ∆7-avenasterol (3.4%) (Figure 1 and Table 2).

### 3.2. Food Intake, Body Weight and Organ Weight

**Figure 2:**
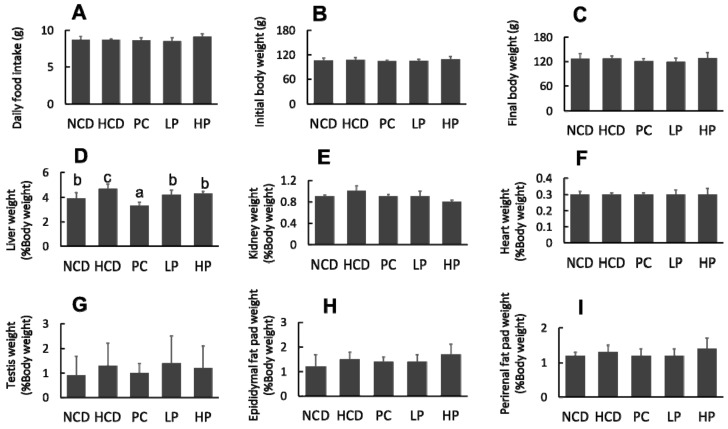
Changes in daily food intake. (**A**), Initial body weight (**B**), final body weight (**C**), relative organ weight of liver (**D**), kidney (**E**), heart (**F**), testis (**G**), epididymal fat pat (**H**) and perirenal fat pat (**I**) in hamsters fed one of the five diets. NCD, no cholesterol diet; HCD, high cholesterol diet containing 0.1% cholesterol; PC, high cholesterol diet containing 0.5% cholestyramine; LP, high cholesterol diet containing a low dose (0.1%) of soybean germ phytosterols (SGP); HP, high cholesterol diet containing a high dose (0.2%) of soybean germ phytosterols (SGP). *n* = 8 for each group. a,b,c = means with different superscript letters differ significantly at *p* < 0.05.

The feeding experiment lasted for six weeks. No significant differences were observed in food intake, mean body weight and organ weights except for the relative liver weight, which was decreased in the LP group and HP group compared with the HCD group during the experimental period (Figure 2).

### 3.3. Plasma Lipoprotein Cholesterol

**Figure 3:**
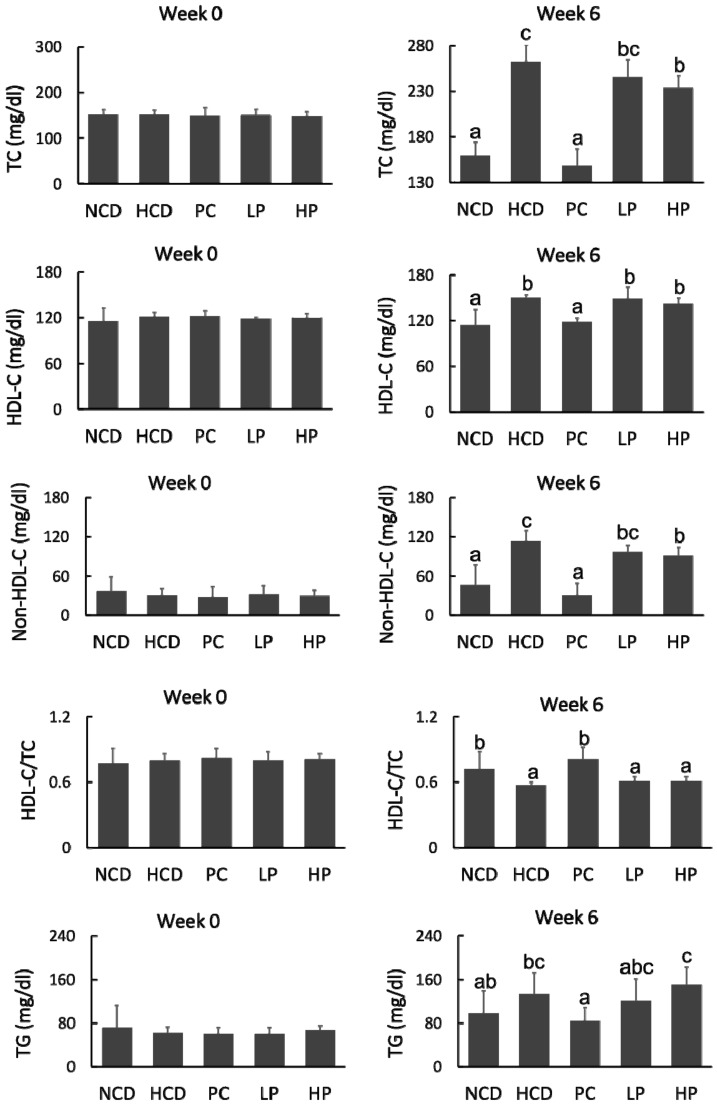
Changes in total cholesterol (TC), high-density lipoprotein cholesterol (HDL-C), non-HDL cholesterol (Non-HDL-C), HDL-C/TC and triglyceride (TG) of hamsters fed one of five diets: NCD, no cholesterol diet; HCD, high cholesterol diet containing 0.1% cholesterol; PC, high cholesterol diet containing 0.5% cholestyramine; LP, high cholesterol diet containing a low dose (0.1%) soybean germ phytosterols (SGP); HP, high cholesterol diet containing a high dose (0.2%) of soybean germ phytosterols (SGP). *n* = 8 for each group. a,b,c = means with different superscript letters differ significantly at *p* < 0.05.

At week 0, the concentrations of plasma TC, HDL-C, non-HDLC and TG among the five groups were at similar levels. After feeding for six weeks, the HCD diet significantly increased plasma TC by 64.6%, compared with the NCD diet, whereas the PC diet prevented plasma TC rise by 43.7% compared with the HCD diet. Feeding the LP and HP diets reduced plasma TC by 6.7–11.1% and non-HDL-C by 14.4–19.7%, respectively, compared with feeding the HCD diet. However, feeding the LP or HP diets had no effect on plasma TG (Figure 3).

### 3.4. Atherosclerotic Plaques

**Figure 4:**
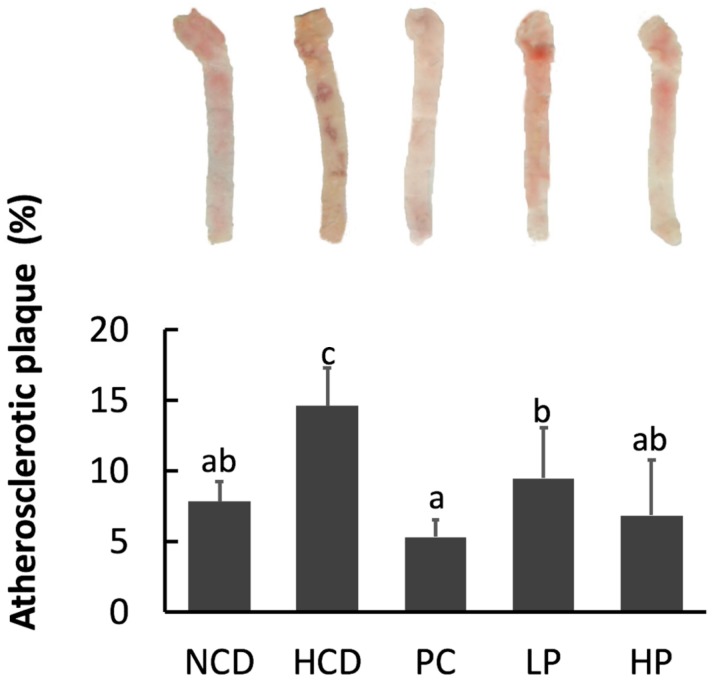
Aorta atherosclerotic plague area in hamsters fed one of the five diets: NCD, no cholesterol diet; HCD, high cholesterol diet containing 0.1% cholesterol; PC, high cholesterol diet containing 0.5% cholestyramine; LP, high cholesterol diet containing a low dose (0.1%) soybean germ phytosterols (SGP); HP, high cholesterol diet containing a high dose (0.2%) of soybean germ phytosterols (SGP). *n* = 8 for each group. a,b,c = means with different superscript letters differ significantly at *p* < 0.05.

Feeding the HCD diet significantly increased the aortic plaque area compared with feeding the NCD diet, while feeding the PC diet effectively reduced the aortic plaque area in comparison with feeding the HCD diet. Feeding the LP and HP diets reduced the atherosclerotic plaque by 35.0–52.9% (Figure 4).

### 3.5. Liver Cholesterol

**Figure 5:**
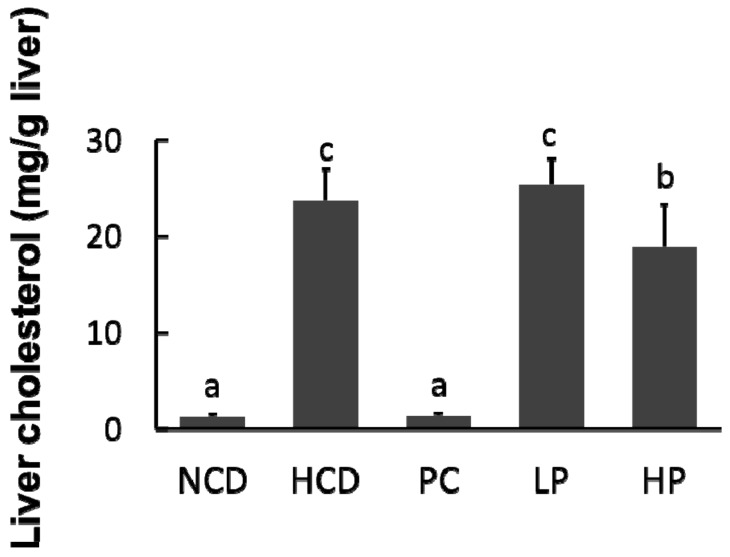
Changes in relative liver cholesterol concentration in hamsters fed one of five diets: NCD, no cholesterol diet; HCD, high cholesterol diet containing 0.1% cholesterol; PC, high cholesterol diet containing 0.5% cholestyramine; LP, high cholesterol diet containing a low dose (0.1%) soybean germ phytosterols (SGP); HP, high cholesterol diet containing a high dose (0.2%) of soybean germ phytosterols (SGP). *n* = 8 for each group. a,b,c = means with different superscript letters differ significantly at *p* < 0.05.

Feeding the HCD diet caused an accumulation of cholesterol in the liver compared with feeding the NCD diet (23.8 mg/g versus 1.3 mg/g). Compared with the HCD diet group, hepatic cholesterol concentration in hamsters fed the LP diet had no significant change, whereas hepatic cholesterol in hamsters fed the HP diet was significantly decreased by 20.8%. Feeding the PC diet containing cholestyramine lowered hepatic cholesterol concentration by 94.2% (Figure 5).

### 3.6. Fecal Total Sterols

**Figure 6:**
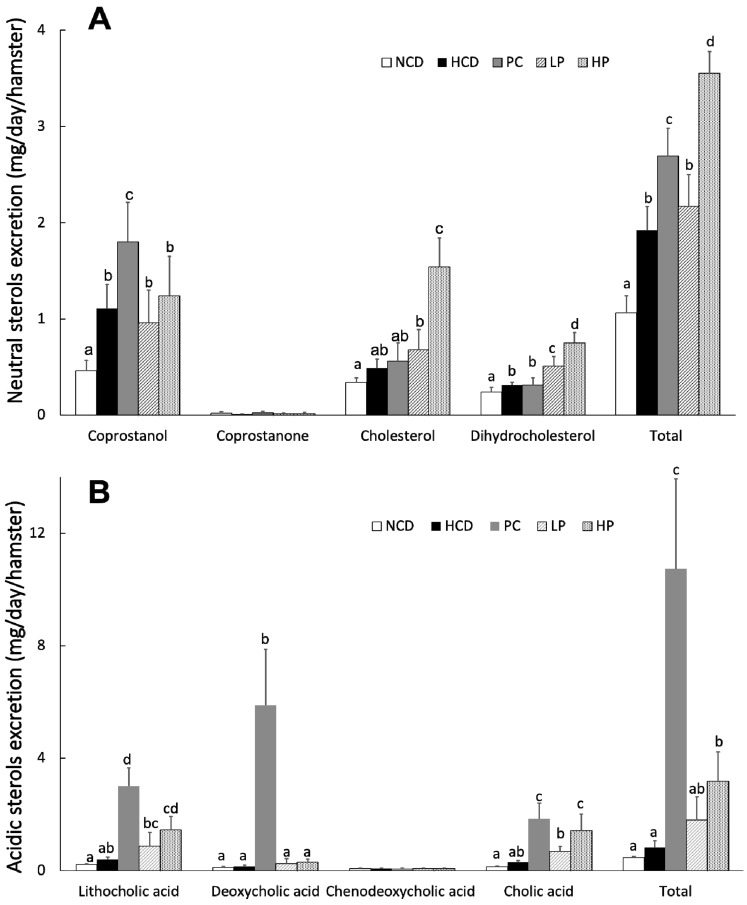
Daily fecal excretion of neutral sterols (**A**) and acidic sterols (**B**) at week 6 in hamsters fed one of five diets: NCD, no cholesterol diet; HCD, high cholesterol diet containing 0.1% cholesterol; PC, high cholesterol diet containing 0.5% cholestyramine; LP, high cholesterol diet containing a low dose (0.1%) soybean germ phytosterols (SGP); HP, high cholesterol diet containing a high dose (0.2%) soybean germ phytosterols (SGP). *n* = 8 for each group. a,b,c = means with different superscript letters differ significantly at *p* < 0.05.

**Figure 7:**
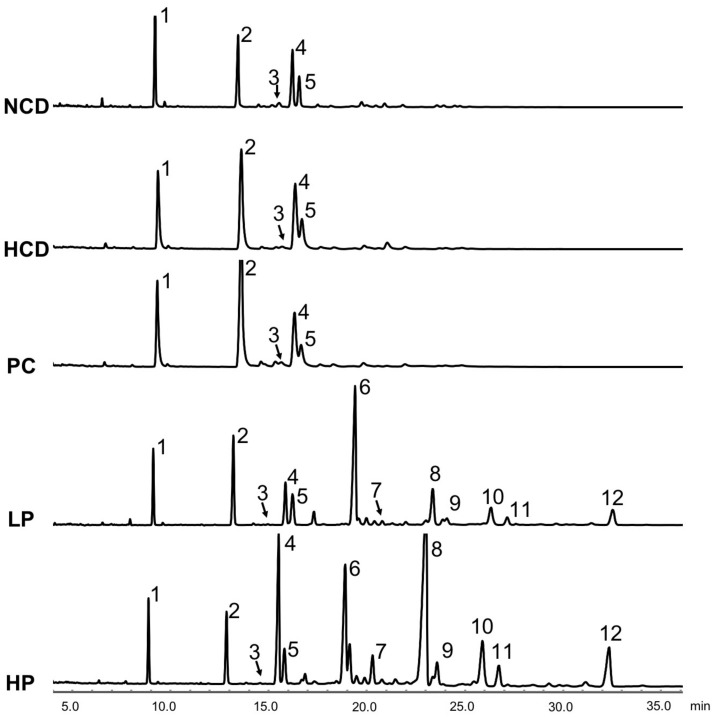
Gas chromatogram of fecal neutral sterols and phytosterols at week 6 in hamsters fed one of five diets: NCD, no cholesterol diet; HCD, high cholesterol diet containing 0.1% cholesterol; PC, high cholesterol diet containing 0.5% cholestyramine; LP, high cholesterol diet containing a low dose (0.1%) soybean germ phytosterols (SGP); HP, high cholesterol diet containing a high dose (0.2%) of soybean germ phytosterols (SGP). Peaks: 1, 5α-cholestane (internal standard); 2, coprostanol; 3, coprostanone; 4, cholesterol; 5, dihydrocholesterol; 6, campesterol; 7, stigmasterol; 8, β-sitosterol; 9, stigmastanol; 10, ∆7-stigmastenol; 11, ∆7-avenasterol; 12, citrostadienol.

Results showed that feeding the LP diet slightly increased the excretion of total fecal neutral sterols (13.0%) while feeding the HP diet markedly increased the excretion of total fecal neutral sterols (84.9%) compared with feeding the HCD diet. Feeding the LP and HP diets could effectively enhance the excretion of total bile acids by 118.3% and 287.8%, respectively. Supplementation of cholestyramine in the diet promoted both the fecal excretion of total neutral sterols and total acidic sterols. Additionally, most unabsorbed SGP were excreted in feces (Table 3, Figure 6 and Figure 7).

### 3.7. mRNA and Immunoblot of Intestinal NPC1L1, ABCG5/8, ACAT2 and MTP, and Hepatic SREBP2, LDLR, HMG-CoA-R, CYP7A1 and LXRα

**Figure 8:**
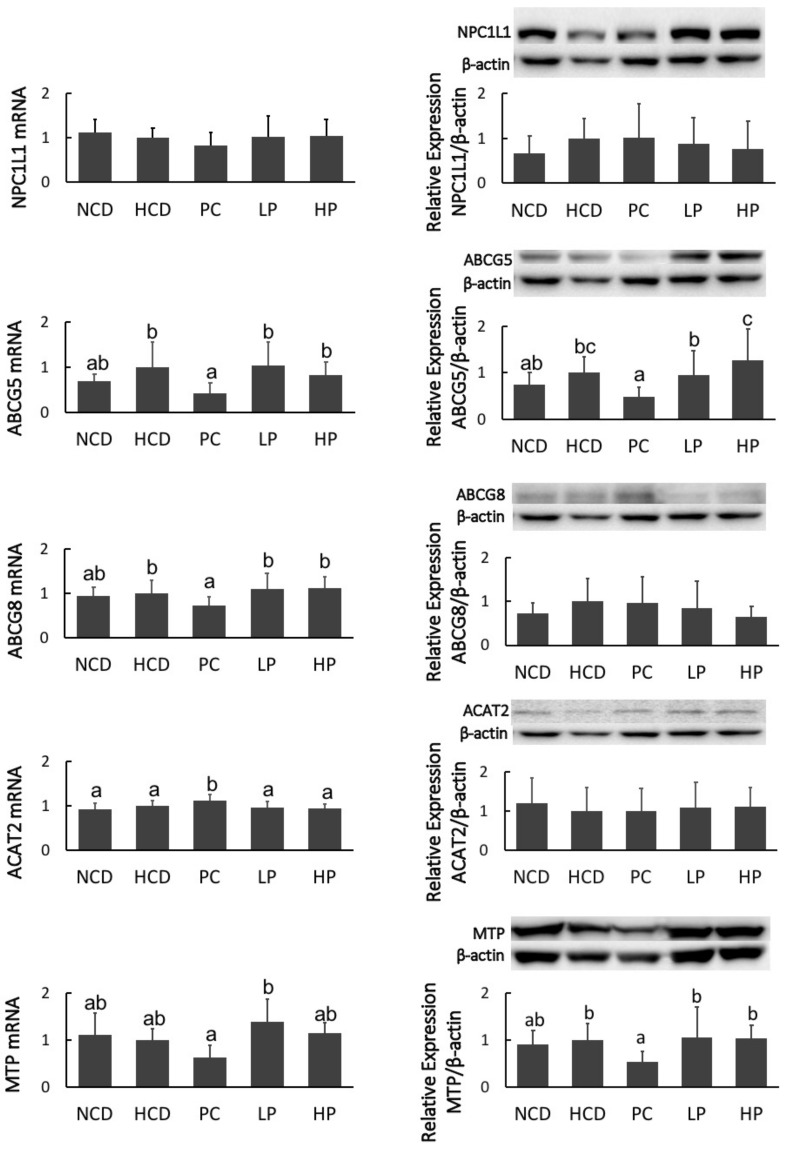
mRNA levels and protein mass of Niemann-Pick C1–like 1 protein (NPC1L1), members 5 and 8 of the ATP binding cassette transporter subfamily G (ABCG5/8), acyl-CoA: cholesterol acyltransferase 2 (ACAT2), and microsomal triacylglycerol transport protein (MTP), in hamsters fed one of five diets: NCD, no cholesterol diet; HCD, high cholesterol diet containing 0.1% cholesterol; PC, high cholesterol diet containing 0.5% cholestyramine; LP, high cholesterol diet containing a low dose (0.1%) soybean germ phytosterols (SGP); HP, high cholesterol diet containing a high dose (0.2%) soybean germ phytosterols (SGP). *n* = 8 for each group. a,b,c = means with different superscript letters differ significantly at *p* < 0.05.

**Figure 9:**
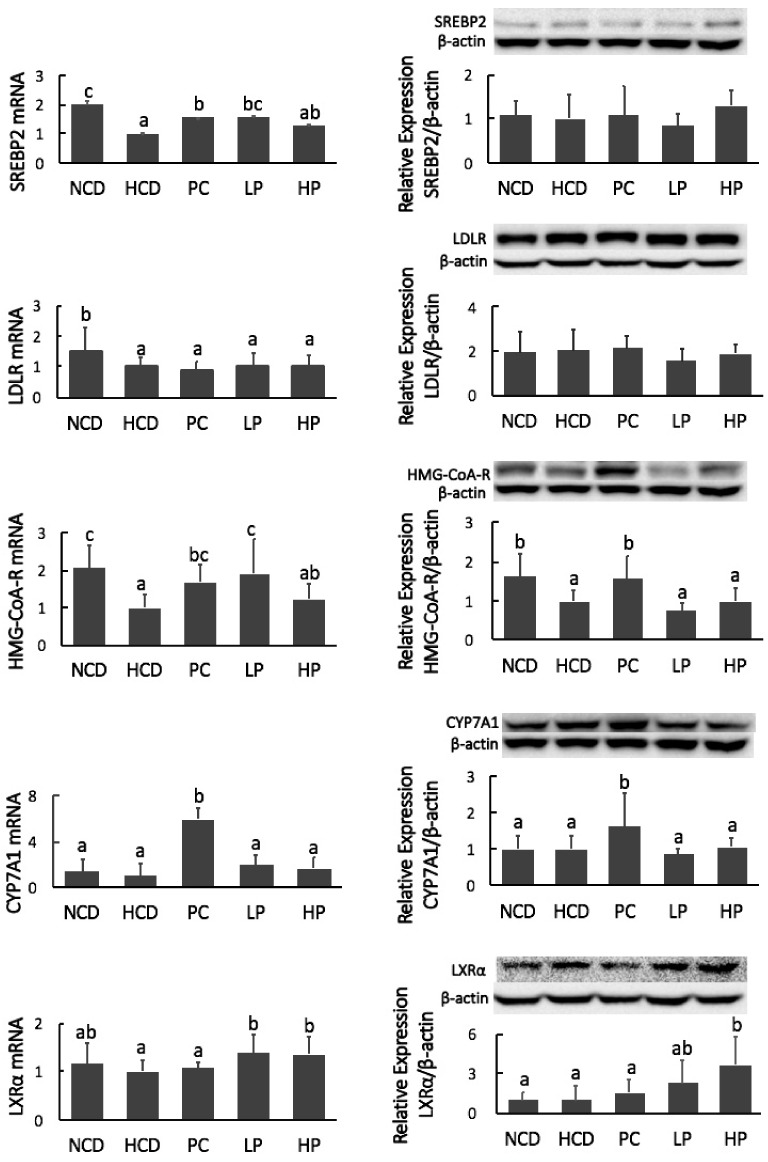
mRNA levels and protein mass of hepatic sterol regulatory element-binding protein-2 (SREBP2), low-density lipoprotein receptor (LDL-R), 3-hydroxy-3-methylglutary-CoA reductase (HMG-CoA-R), cholesterol-7a-hydroxylase (CYP7A1) and liver X receptor alpha (LXRα) in hamsters fed one of five diets: NCD, no cholesterol diet; HCD, high cholesterol diet containing 0.1% cholesterol; PC, high cholesterol diet containing 0.5% cholestyramine; LP, high cholesterol diet containing a low dose (0.1%) soybean germ phytosterols (SGP); HP, high cholesterol diet containing a high dose (0.2%) of soybean germ phytosterols (SGP). *n* = 8 for each group. a,b,c = means with different superscript letters differ significantly at *p* < 0.05.

Feeding the PC diet containing cholestyramine downregulated mRNA levels of ABCG8 and MTP, and decreased the protein mass of MTP, while it upregulated mRNA levels of ACAT2. However, feeding the LP and HP diets had no effect on the mRNA level or protein masses of NPC1L1, ABCG5/8, ACAT2 and MTP (Figure 8). Hamsters fed the PC diet had greater mRNA expression and protein mass of CYP7A1. Feeding the PC diet also significantly increased mRNA expression of HMG-CoA-R. Feeding the LP diet upregulated the mRNA level of SREBP2 and HMG-CoA-R without affecting their protein masses. Feeding the HP diet significantly increased the gene expression of LXRα at both the mRNA and protein levels. However, no significant differences in the mRNA expression or protein abundance of SREBP2, LDLR, HMG-CoA-R and CYP7A1 were observed between the HCD and HP diet groups (Figure 9).

### 3.8. Displacing Effects of SGP on Incorporation of Cholesterol into Micelles

**Figure 10:**
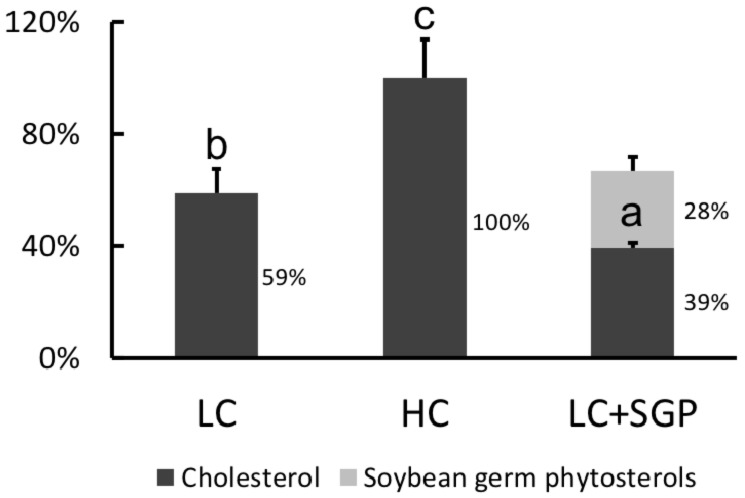
Effect of soybean germ phytosterols (SGP) on the incorporation of cholesterol into micelles. The three micellar solutions were prepared by adding cholesterol and soybean germ phytosterol into the basal micellar solution: LC, 0.25 mM cholesterol; HC, 0.5 mM cholesterol; LC + SGP, a binary mixture of 0.25 mM cholesterol and 0.25 mM soybean germ phytosterols (SGP). Data were expressed as mean ± SD, *n* = 6, with the amount of cholesterol in CH micelles being arbitrarily regarded as 100%. a,b,c = means with different superscript letters differ significantly at *p* < 0.05.

Cholesterol in HC micelles (0.50 mM) was arbitrarily regarded as 100%. Results showed that the incorporation of cholesterol in LC micelles (0.25 mM) was reduced to 59.0%. When 0.25 mM SGP was added into LC micelles, the cholesterol incorporation was reduced to 39.0%. Meanwhile, incorporation of SGP into micelles was 28.0%, indicating that SGP could displace the cholesterol from micelles (Figure 10).

## 4. Discussion

SGP is rich in soybean germ oil and contains eight phytosterol species. The present study was the first of its kind to investigate the effect of SGP on blood cholesterol and the associated underlying mechanisms in hamsters with hypercholesterolemia. As a herbal extract, SGP was effective in improving the lipoprotein cholesterol profiles in hamsters fed a high cholesterol diet. In this study, dietary SGP at doses of 0.1% and 0.2% could effectively decrease plasma TC by 6.7–11.1% and non-HDL-C by 14.4–19.7%, respectively (Figure 3).

Hypercholesterolemia is a risk factor for inducing atherosclerosis. In the present study, SGP dose-dependently reduced the formation of atherosclerotic plaque area by 35.0–52.9% (Figure 4), which was in agreement with previous research in hamsters given β-sitosterol [17,25]. Results showed that SGP was comparable to cholestyramine in preventing the formation of atherosclerotic plaques, even though its effect on plasma cholesterol level was weaker than that of cholestyramine. One possible reason is that high blood cholesterol is only one of many factors, such as inflammation, oxidative stress, and vascular dysfunction, for inducing atherosclerosis.

We propose that the blood cholesterol-lowering activity of SGP is partially mediated by inhibiting cholesterol absorption. Cholesterol in the intestine is derived from two sources, namely diet and bile fluids [26]. Fecal cholesterol is usually used as an index of cholesterol absorption. It was evident that the addition of SGP in the diet at 0.1% and 0.2% could markedly increase the excretion of cholesterol by 38.8–214.3% (Figure 6). As most unabsorbed cholesterol is converted to its microbial metabolites coprostanol, coprostanone and other related neutral sterols in the large intestine, total neutral sterols in feces are a better biomarker of total cholesterol excretion [27]. The fecal analysis found that SGP could increase the excretion of total fecal neutral sterols by 13.0–84.9% (Figure 6 and Figure 7), proving that SGP was effective in reducing cholesterol absorption in the intestine. Cholesterol absorption in the intestine requires several transporters and enzymes, including NPC1L1, ACAT2, MTP and ABCG5/8. The present study found that SGP had no effect on the gene expression of these transporters and enzymes (Figure 8). Instead, SGP could effectively displace the cholesterol from micelles (Figure 10). It was therefore concluded that SGP could inhibit cholesterol absorption via displacing cholesterol from micelles rather than affecting the genes involved in cholesterol absorption at doses of 0.1–0.2% in hamsters.

The blood cholesterol-lowering activity of SGP is also partially mediated by its ability to increase the excretion of fecal bile acids. The production of bile acids is one of the major mechanisms that acts to remove and eliminate excess cholesterol in mammals via the bile duct to the intestine [28]. The primary bile acids, cholic acid and chenodeoxycholic acid, are synthesized in the liver, while the secondary bile acids, deoxycholic acid and lithocholic acid, are microbial metabolites of the primary bile acids in the large intestine. The effect of phytosterols on bile acid excretion is still inconclusive. Some studies proved that phytosterols promote bile acid excretion in hamsters, while others reached the opposite conclusion in mice [17,25,29,30]. The discrepancies in different studies is likely due to the different animal models used. Hamsters are generally considered as a better model than other rodents because they excrete bile acids in a manner similar to that of humans. In this study, SGP consumption markedly enhanced the excretion of primary and secondary bile acids as a whole by 118.3% and 287.8%, respectively, suggesting that SGP supplementation reduced blood cholesterol levels by a mechanism of enhancing the excretion of not only cholesterol but also bile acids (Figure 6).

The liver is the most important organ for maintaining cholesterol homeostasis. We examined the hepatic genes governing cholesterol homeostasis. Results demonstrated that SGP did not affect gene expression of SREBP2, HMG-CoA-R, LDLR, and CYP7A1 at the translational level (Figure 9), suggesting that the SGP-induced reduction of blood cholesterol is unlikely mediated via regulation of these genes because phytosterols are poorly absorbed and the amount of phytosterols reaching the liver is very minimum [31]. CYP7A1 is essential to convert cholesterol to bile acids, and thus the effects of SGP on bile acid synthesis may be minimal. One possible explanation for the interaction of SGP with bile acid excretion was that large amounts of SGP in the small intestine may inhibit not only cholesterol absorption but also bile acid reabsorption [32].

Trans-intestinal cholesterol efflux (TICE) is recognized as a new significant alternative route to the hepatobiliary system. It has been demonstrated that TICE accounts for 35% and 70% of fecal neutral sterol excretion in humans and mice, respectively [33,34]. ABCG5/8 are responsible for cholesterol efflux in enterocytes [35,36]. The present study observed that SGP did not have any significant effect on the gene expression of these transporters and enzymes. Another important nuclear receptor involved in TICE is the liver X receptor. van der Veen et al. proved that TICE was stimulated upon LXRα activation [37]. In the present study, SGP supplementation significantly upregulated the gene expression of LXRα in both mRNA and protein levels. Additionally, fecal neutral sterol excretion is regarded as a surrogate marker of TICE. Combining them together, we hypothesized that the plasma cholesterol-lowering activity of SGP was also likely mediated by promoting neutral sterol excretion via activating TICE through gene upregulation of LXRα [33]. 

The doses of SGP used in the present study are physiologically achievable. Supplementation of 0.1% and 0.2% SGP into diets was equivalent to 0.5–1 g/2000 kcal. It has been recommended that the daily consumption of 2 g of phytosterols can effectively lower plasma TC by 9–14% in humans [9]. Assuming that the daily caloric intake for an adult human is about 2000 kcal, the HP diet could provide 1 g of SGP per day. In this regard, SGP was highly effective as a nutraceutical in reducing blood cholesterol level if the data in hamsters can be extrapolated to humans.

## 5. Conclusions

In summary, SGP as a herbal medicine could remarkedly reduce plasma TC and non-HDL-C. The cholesterol-lowering activity of SGP was mediated by inhibiting cholesterol absorption and promoting fecal sterol excretion.

## Figures and Tables

**Table 1:** Composition of the five diets. NCD, no cholesterol diet; HCD, high cholesterol diet containing 0.1% cholesterol; PC, high cholesterol diet containing 0.5% cholestyramine; LP, high cholesterol diet containing a low dose (0.1%) of soybean germ phytosterols (SGP); HP, high cholesterol diet containing a high dose (0.2%) of soybean germ phytosterols (SGP).

Ingredients (g/1000 g Diet)	NCD	HCD	PC	LP	HP
Corn starch	485	484	479	483	482
Casein	230	230	230	230	230
Sucrose	113	113	113	113	113
Lard	95	95	95	95	95
Mineral mixture	38	38	38	38	38
Vitamin mixture	19	19	19	19	19
Gelatin	19	19	19	19	19
DL-Methionine	1	1	1	1	1
Cholesterol	0	1	1	1	1
Cholestyramine			5		
Soybean germ phytosterols	0	0	0	1	2

**Table 2:** Composition of soybean germ phytosterols extract (SGP).

SGP Composition	% Total
Ergosterol	0.5
Campesterol	7.2
Stigmasterol	4.8
β-sitosterol	60.0
Stigmastanol	4.7
∆7-stigmastenol	9.8
∆7-avenasterol	3.4
Citrostadienol	7.4
Others	2.2

**Table 3:** Daily fecal excretion of phytosterols at week 6 in hamsters fed one of the five diets: NCD, no cholesterol diet; HCD, high cholesterol diet containing 0.1% cholesterol; PC, high cholesterol diet containing 0.5% cholestyramine; LP, high cholesterol diet containing a low dose (0.1%) soybean germ phytosterols (SGP); HP, high cholesterol diet containing a high dose (0.2%) soybean germ phytosterols (SGP).

	NCD	HCD	PC	LP	HP	*p* Value
Phytosterols (mg/day/hamster)						
Campesterol	ND	ND	ND	2.13 ± 0.56	3.64 ± 0.86	NA
Stigmasterol	ND	ND	ND	0.07 ± 0.02	0.21 ± 0.07	NA
β-sitosterol	ND	ND	ND	0.74 ± 0.20	3.02 ± 0.95	NA
Stigmastanol	ND	ND	ND	0.12 ± 0.06	0.16 ± 0.04	NA
∆7-stigmastenol	ND	ND	ND	0.33 ± 0.05	0.81 ± 0.09	NA
∆7-avenasterol	ND	ND	ND	0.11 ± 0.02	0.26 ± 0.03	NA
Citrostadienol	ND	ND	ND	0.35 ± 0.07	0.97 ± 0.24	NA
Total	ND	ND	ND	3.86 ± 0.45	9.09 ± 0.90	NA

ND = not detectable, *n* = 8 for each group. a,b,c = means with different superscript letters differ significantly at *p* < 0.05.
